# Global sea-surface iodide observations, 1967–2018

**DOI:** 10.1038/s41597-019-0288-y

**Published:** 2019-11-26

**Authors:** Rosie J. Chance, Liselotte Tinel, Tomás Sherwen, Alex R. Baker, Thomas Bell, John Brindle, Maria Lucia A. M. Campos, Peter Croot, Hugh Ducklow, He Peng, Frances Hopkins, Babette Hoogakker, Claire Hughes, Timothy D. Jickells, David Loades, Dharma Andrea Reyes Macaya, Anoop S. Mahajan, Gill Malin, Daniel Phillips, Ieuan Roberts, Rajdeep Roy, Amit Sarkar, Alok Kumar Sinha, Xiuxian Song, Helge Winkelbauer, Kathrin Wuttig, Mingxi Yang, Zhou Peng, Lucy J. Carpenter

**Affiliations:** 1 Wolfson Atmospheric Chemistry Laboratories, Department of Chemistry, University of York, York, UK; 2 National Centre for Atmospheric Science (NCAS), Department of Chemistry, University of York, York, UK; 3 Centre for Ocean and Atmospheric Sciences, School of Environmental Sciences, University of East Anglia, Norwich Research Park, Norwich, NR4 7TJ UK; 4 Plymouth Marine Laboratory, PL1 3DH Plymouth, UK; 5 Departamento de Química, FFCLRP, Universidade de São Paulo (USP), Ribeirão Preto, SP 14040-901 Brazil; 6 School of Natural Sciences, National University of Ireland Galway (NUI Galway), H91 TK33 Galway, Ireland; 7 Earth & Environmental Sciences, Columbia University, PO Box 1000, Palisades, New York 10964-8000 USA; 8 State Key Laboratory of Geohazard Prevention and Geoenvironment Protection, Chengdu University of Technology, Chengdu, 610059 China; 9 School of Environment, Chengdu University of Technology, Chengdu, 610059 China; 10 School of Energy, Geoscience, Infrastructure and Society, Heriot-Watt University, EH14 4AS Edinburgh, UK; 11 Department of Environment and Geography, University of York, Wentworth Way, Heslington, York YO10 5NG UK; 12 Centre for Marine Environmental Sciences, Universität Bremen, 28359 Bremen, Germany; 13 Centre for Climate Change Research, Indian Institute of Tropical Meteorology (IITM), Pune, India; 14 National Remote Sensing Centre, Indian Space Research Organisation, Hyderabad, India; 15 National Centre Polar and Ocean Research, Vasco-da-Gama, Goa 403 804 India; 16 Ecosystem based management of marine resources, (EBMMR), Environment and Life Sciences Research Center, Kuwait Institute for Scientific Research, Salmiya, Kuwait; 17 Key Laboratory of Marine Ecology and Environmental Sciences, Institute of Oceanology, Chinese Academy of Sciences, 7 Nanhai Road, Qingdao, 266071 China; 18 GEOMAR Helmholtz Centre for Ocean Research Kiel, 24015 Kiel, Germany; 19 Antarctic Climate and Ecosystems Cooperative Research Centre (ACE CRC), University of Tasmania, Private Bag 80, Hobart, TAS 7001 Australia

**Keywords:** Element cycles, Atmospheric chemistry, Marine chemistry

## Abstract

The marine iodine cycle has significant impacts on air quality and atmospheric chemistry. Specifically, the reaction of iodide with ozone in the top few micrometres of the surface ocean is an important sink for tropospheric ozone (a pollutant gas) and the dominant source of reactive iodine to the atmosphere. Sea surface iodide parameterisations are now being implemented in air quality models, but these are currently a major source of uncertainty. Relatively little observational data is available to estimate the global surface iodide concentrations, and this data has not hitherto been openly available in a collated, digital form. Here we present all available sea surface (<20 m depth) iodide observations. The dataset includes values digitised from published manuscripts, published and unpublished data supplied directly by the originators, and data obtained from repositories. It contains 1342 data points, and spans latitudes from 70°S to 68°N, representing all major basins. The data may be used to model sea surface iodide concentrations or as a reference for future observations.

## Abstract (summary)

**Table:** 

Measurement(s)	iodide
Technology Type(s)	digital curation
Factor Type(s)	sampling time and place
Sample Characteristic - Environment	sea water • ocean
Sample Characteristic - Location	Earth (planet)

Machine-accessible metadata file describing the reported data: 10.6084/m9.figshare.10130129

## Background & Summary

There has recently been a resurgence of interest in the marine iodine cycle, reflecting its involvement in a diverse range of processes, from influencing air quality (e.g.^1^) to recording ocean deoxygenation in sediments (e.g.^2^). Iodine is a redox active element that is present in seawater in two main forms, iodide (I^−^) and iodate (IO_3_^−^). Sea-to-air transfer is the dominant source of iodine to the atmosphere, where it is subject to atmospheric processing prior to deposition back to the sea or onto land. It is an essential nutrient for many organisms including humans, and deficiency in humans leads to goitre, cretinism and is the leading cause of preventable mental retardation globally^3^. Iodine radionuclides are also released to the oceans by anthropogenic activities, where they will be subject to the same processes of biogeochemical cycling and volatilisation as the naturally occurring stable isotope^4^. Despite the wide ranging impacts of iodine biogeochemistry, the distribution of iodine species in the oceans remains relatively poorly understood. Here we present an updated compilation of all currently available sea surface iodide concentrations. The data set is specifically intended to inform studies of the sea-air exchange of iodine species, but may also be of use in improving understanding of the marine iodine cycle more generally.

The reaction of iodide with ozone at the surface of the ocean has been established as an important sink for ozone, thought to be responsible for around one third of the total ozone loss by dry deposition^5^. The reaction liberates reactive iodine compounds to the atmosphere, which in turn contribute to further ozone removal processes. Gas phase reactions involving iodine are estimated to account for up to 15% of tropospheric ozone losses^6^. To incorporate this chemistry, global and regional air quality and atmospheric chemistry models have begun to include predicted sea-surface iodide fields derived from parameterisations (e.g.^5,7–9^). However, current sea surface iodide parameterisations are known to have biases^10^, are subject to substantial uncertainty^6^, and do not take advantage of recent and substantial increases in the number of available observations (e.g.^11^).

**Fig. 1:**
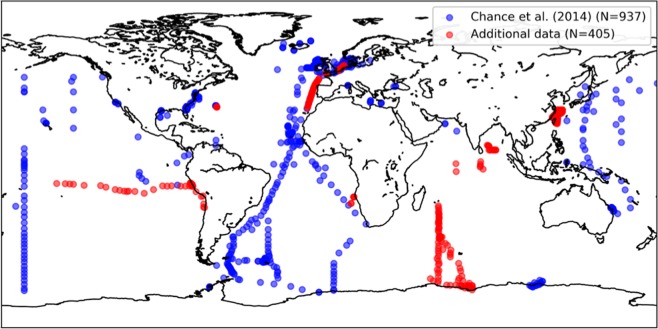
Locations of iodide observations included in our dataset. New data reported here is in red and existing data from Chance *et al*.^12^ is blue. Figure produced with Python Matplotlib^79^.

The observational data underpinning iodide parameterisations is sparse, and has hitherto not been publicly available in a collated form. In many cases, iodide observations are not readily accessible in a digital form (i.e. are only presented in graphical format). To facilitate the development and validation of improved sea surface iodide parameterisations, we have compiled all available sea surface iodide observations. The dataset is an extended version of that used in our earlier publication^12^, in which we described the large scale sea surface iodide distribution and presented correlations between iodide and other oceanographic variables, but did not publish the observations themselves. The dataset we now present incorporates more than 400 new observations (see Fig. 1), including new, basin scale transects from the Indian Ocean (currently unpublished) and the tropical eastern Pacific^11^, both of which were previously undersampled^12^. This new extended dataset is freely available via the British Oceanographic Data Centre (BODC; http://doi.org/czhx)^13^.

We anticipate that the primary use of the dataset will be modelling of ozone deposition to the sea surface and/or associated trace gas emissions to the atmosphere. It has been used to generate new monthly parameterised sea-surface iodide fields (12 × 12 km resolution) using a machine learning approach, described in our accompanying partner publication^10^. The dataset may also be of interest in other areas of iodine research. In particular, improved understanding of the marine iodine cycle is needed to refine the use of iodine speciation as a paleo-oceanographic tracer of past ocean oxygenation (e.g.^2^), and to better predict the impacts of iodine radionuclides released to the environment by anthropogenic activities (e.g.^4^).

## Methods

### Data compilation

The data set includes iodide measurements made by a number of different research groups (Online-only Table 1). These were collated from the following sources:

A. *Published manuscripts*. Data was digitised from tables and graphics, either by hand or using the free online tool WebPlotDigitizer (https://automeris.io/WebPlotDigitizer).
B. *Data originators*. Data (both published and unpublished) was provided directly by the owners.
C. *Data repositories*. Data was obtained by request or on-demand download from hosting repositories (e.g. BODC, PANGAEA, the US JGOFS Data System).

Following the approach adopted previously^12^, ‘surface’ concentrations are considered to be those from depths of less than 20 m. As discussed in Chance *et al*.^12^, the ocean is usually considered well mixed to this depth, and to restrict ‘surface data’ to shallower depths would substantially reduce the number of observations included. We examined a sub-set of data (n = 93) where observations were available from multiple depths within the upper 20 m of the water column. While significant differences were occasionally found between individual pairs of samples collected from depths of ~1-2 m and ~10 m at a given station, concentrations were within 10 nM in almost 50% of pairs (49.5%), and 80% were within 26 nM. Statistical analysis (using a paired students t-test) found no significant difference between samples from different depths within the upper 20 m. The exact depth of near surface samples can itself have high relative uncertainty, as factors such as sea swell can lead to metre scale fluctuations to the exact depth of e.g. a ship seawater inlet. Furthermore, the exact depths of such inlets, or the ‘surface’ sample bottle, was not always stated in the original data sources. Therefore, we have not included depth as a parameter in our compiled data set and no distinction has been made between samples obtained using a CTD rosette fitted with Niskin bottles (or similar), a pumped underway seawater supply or a manual method (such as bucket sampling).

Each data set was entered onto an individual Excel spreadsheet in a standard format. Rarely, source values were below the limit of detection (LoD) for the method used. Where this was the case, we have used a substitute value of 0.75 x the estimated LoD and the data point was flagged (column ‘ErrorMethod’). No further processing has been applied to any of the data. It has not been normalised e.g. to salinity. Required fields from individual ‘input’ files were then collated into a single comma-separated value (.csv) file using open-source Python code, including the Pandas package^14^.

A total of 1342 observations, from 57 individual data sets has been collated (Online-only Table 1). This is an increase of 417 observations (45%) on that included in our earlier compilation^12^. Locations of individual data points are shown in Fig. 1, which highlights how the expanded dataset increases spatial coverage. The earliest observations were made in 1967 and the most recent in 2018. For some data points (n = 32) the date of sampling is not specified as this was not given in the original publication. Ten of the input data sets are currently unpublished.

### Additional fields

Each iodide observation is associated with the record fields listed in Table 1. In addition to spatial and temporal co-ordinates, the estimated uncertainty and analytical method used to generate the observations are provided.

**Table 1:** Data record fields *or* Column names, column description and units for each field included in the sea surface iodide database.

Field header	Description & unit or key
Data_Key Data_Key_id	Unique identifier for dataset. Unique identifier for the data point: Dataset (see above) followed by the index of the datapoint in the input dataset
Latitude Longitude Year Month Day	Latitude coordinate of data point, decimal degrees north Longitude coordinate of data point, decimal degrees east Sometimes available Sometimes available Rarely available
Iodide Iodide error Error Flag	Iodide concentration, nmol L^−1^ Estimated uncertainty on iodide concentration, nmol L^−1^ Indicates way uncertainty was estimated: 1 Precision stated in paper or by source, based on replicate analyses of selected samples 2 Precision assumed same as in similar work using same method 3 Individual samples all analysed in replicate, uncertainty is range (n = 2) or sd (n > = 3) 4 Propagated analytical uncertainty for replicates of a given sample, where this is greater than uncertainty determined by (3) 5 Analytical uncertainty derived from scatter in repeat scans within a single analysis 6 Propagation of stated uncertainties on Iodate and TII, where [I^−^] calculated by difference 7 Value < LoD; replaced with substitute value 8 None given & no comparable methods 9 Standard deviation of similar samples that have been grouped to give an average iodide concentration for the position
Method	See Table 2 for method codes
Coastal LocatorFlag	0 = open ocean location 1 = coastal location Indicates way coastal flag was assigned as follows: 0 Location found by province picker, coastal determined according to Province 1 Location not found by Province picker as too close to coast, so Province manually assigned. Province necessarily coastal. 2 Province is open ocean, but individual samples known to be coastal e.g. Bermuda Inshore
Reference	Publication in which the data set is described.

#### Method

Analytical methods are summarised in Table 2. In the majority of cases (~53%), iodide was measured by cathodic stripping square wave voltammetry (CSSWV) according to the method of Campos^15^. However a range of other measurements techniques were also used. Iodide was sometimes measured as the difference between the total inorganic iodine (TII) concentration and the iodate concentration.

**Table 2:** Analytical methods and associated uncertainties.

Type	Key	Analytical Method(s)	Method Reference(s)	Typical Uncertainty	n data sets	n data points
Difference [I^−^] = [TII] − [IO_3_^−^] *Where TII is total inorganic iodine*	1	*Spectrophotometric:* a. IO_3_^−^ by spectrophotometric detection of I_3_^−^ b. TII by catalytic effect on Ce^IV^ and As^III^ reaction, with spectrophotometric determination of Ce^IV^ *OR* c. TII by oxidation + method (a) *OR* d. Estimate TII from deep-water IO_3_^−^ average, only^26^	^27,28^ ^29^ ^18^	*Propagate errors on IO*_*3*_^−^ *& TII* a. IO_3_^−^ error ~1%^27,30^, or 13^31^ – 20 nM^18^ b. TII error ~0.06%^30^ or 6 nM^31^ c. TII error ~ 20 nM^18^	11	382
	2	*Differential Pulse Polarography (DPP)* a. IO_3_^−^ by DPP b. TII by oxidation + method (a)	^32,33^	*Propagate errors on IO*_*3*_^−^ *& TII* a. IO_3_^−^ error ~2.5–5%^34,35^ b. TII error ~2.5–5%^34,35^	3	48
Electrochemical	3	*Cathodic Stripping Square Wave Voltammetry (CSSWV)*	^15,36^	2–10%;^15,16,36–38^ frequently quoted as ≤5%	30	706
	4	*Differential Pulse Cathodic Stripping voltammetry* (sulphite removal of oxygen)	^39^	1%^40^	1	1
	5	*Automated flow through electrode*	^41^	~5% or 10 nM in artificial seawater^23^	1	13
Chromatographic	6	*Anion exchange* + *precipitation*, *neutron activation analysis (NAA)*	^42^	5%^42^	2	7
	7	*Anion exchange* + *Inductively coupled plasma mass spectrometry (ICP-MS)*	^43–45^	3–7%^43,45^	4	90
	8	*Anion exchange High Performance Liquid Chromatography (HPLC) with spectrophotometric detection*	^46^	~5%^46^	1	7
	9	*Matrix elimination ion chromatography with spectrophotometric detection*	^47^	2%^47^	1	13
Electrokinetic	10	*Capillary electrophoresis*	^48^	~3%^49^	1	3
Precipitation	11	*Ag Precipitation* + *spectrophotometry*	^22,24^	3–5%^20,24^	2	70
	13	*Pd precipitation* + *NAA*	^50^	~5% (13 nM)^50^	1	2

#### Uncertainty

**Fig. 2:**
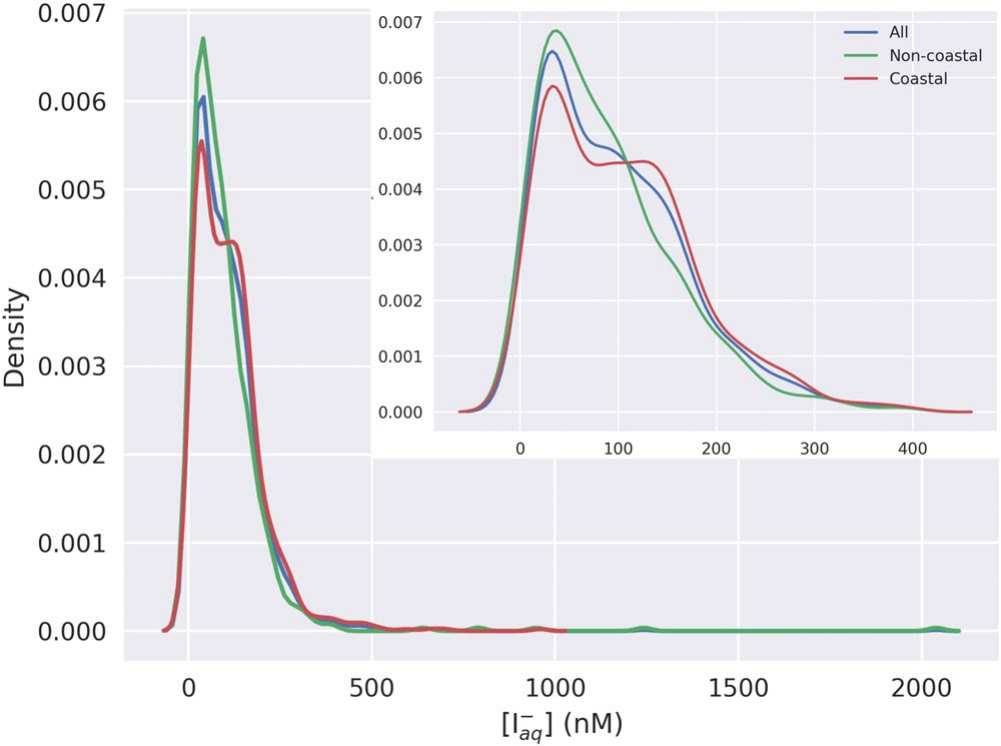
Estimated probability density function (PDF; kernel density estimate) for sea surface iodide observations. Plot shows all data (blue) combined, and open ocean (green) and coastal (red) data treated separately. Expanded inset shows values <400 nM only. Figure produced with Python Matplotlib^79^ and Seaborn^80^ packages.

Measurements of iodide in seawater are subject to non-trivial analytical uncertainties, which should be considered when using the data set. An estimate of the uncertainty associated with each observation has been included, using either information provided by the data source where available, or comparison to other measurements using the same analytical method. The uncertainty estimates provided are typically derived from replicate analyses of the same sample, and so represent the precision of the measurements. As insufficient information was available to quantify the precision in the same way for all observations, the approach used to estimate the precision is also included (see Table 1). Relative uncertainty estimates for each analytical method, for typical ambient concentrations in a seawater matrix, are also provided in Table 2. The precision given for each data set is often 5% (Table 2), which reflects the stated repeatability of the CSSWV method^15^ and a number of other measurements used. However, we note that repeat analyses of samples using this method can sometimes give lower precision (e.g. ~10%)^16^. Considering all data points in our dataset, we find ~75% have a precision of 10% or less, and ~51% have an precision of 5% or less. Such uncertainties are modest in comparison to the global scale variation in sea surface iodide concentrations (from less than 10 to more than 200 nM; Fig. 2).

As the uncertainty estimates provided are typically derived from replicate analyses of the same sample they only estimate the short (days) to medium term (approx. monthly) repeatability. A fuller consideration of the uncertainty should also include the longer term (months to years) reproducibility, and an estimate of any uncertainties arising from bias, and thus may result in a larger uncertainty value. These sources of uncertainty are as yet poorly documented for the determination of iodide in seawater. At least in the case of the most commonly used method (CSSWV), we believe the contribution of long term reproducibility and bias to be small relative to the short-term precision. This is because the key of sources of uncertainty (e.g. that associated with making standard additions and sample dilutions by pipette, or variation in mercury electrode drop size) operate over a short time scale. Within our own laboratory, we have been monitoring long term reproducibility of the CSSWV method using aliquots of a near shore seawater sample, and estimate it at ~12% RSD over a period of 11 months (analysis by three operators using two different instruments; individual aliquots stored at −20 °C and defrosted within a few days of analysis), compared to ~monthly repeatability of 7–12% and repeatability over a few days of 5 to 18%. Changes taking place during storage will also contribute to the overall uncertainty of reported observations; for samples stored frozen (−16 °C), average iodide recovery after one year was 95–96%, compared to an average standard deviation of 5–8%^15^. In the majority of data sets we include, samples were stored frozen for less than one year prior to analysis, others were either analysed immediately following collection or stored for a shorter period refrigerated. Therefore we assume that storage artefacts were minimal. This view is supported by the oceanographic consistency found between stored and freshly analysed samples.

Assessment of bias in iodide in seawater determinations is hindered by the lack of a suitable reference material – many similar reference materials e.g. for trace metals, are acidified, which is unsuitable for the preservation of iodine speciation. Inaccuracy in standard preparation will contribute to bias in the short-term (all samples analysed using same standard), but are likely to become a random error in the longer term (several standards used over time). In either case, this should be a small contribution, as the uncertainty associated with preparing a typical analytical standard (e.g. 10 μM standard) should be less than 1% in a competent lab with well-maintained and calibrated equipment (e.g. balance, pipette). Other contributions to bias, such as matrix effects, are minimised by the use of standard additions rather than external calibration in the CSSWV protocol. In the absence of an iodide reference material, Campos^15^ tested the accuracy of the CSSWV method using solutions of known iodate concentration and a reduction step, and found it to be 99 ± 5.7% for 34 analyses. Given the current interest in marine iodide concentrations^2,10,11^, we believe that an inter-laboratory calibration exercise leading to development of a saline iodide reference material with a consensus value would be very timely. Such an exercise could follow the model of the recent GEOTRACES inter-calibration scheme (http://www.geotraces.org/Intercalibration).

#### Geographical categorisation

Data points are categorised as either ‘coastal’ or ‘non-coastal’. Following the approach used in Chance *et al*.^12^, this is determined by the designation of their static Longhurst biogeochemical province^17^. In most cases, the Longhurst province was assigned automatically, according to the nearest whole number degree of latitude and longitude. For a small number of samples collected very close to the coast, province (and hence coastal/non-coastal) was assigned manually - these samples are flagged (see Table 1). As in Chance *et al*.^12^, a small number of samples collected near Bermuda were also categorised as ‘coastal’ despite being located in an open ocean province (North Atlantic Subtropical Gyre Province (West)), as they were collected from an inshore area^18^. These samples are identified as such in the ‘Locator Method’ column.

#### Ancillary data

Note that original ancillary data such as temperature and salinity is not included, as this was not reliably available for all data sets. Instead we recommend the use of climatological data (e.g. the World Ocean Database and World Ocean Atlas Series) selected according to user needs.

## Data Records

The compiled dataset is hosted by BODC (10.5285/7e77d6b9-83fb-41e0-e053-6c86abc069d0)^13^, and is available as a single.csv file (plus a separate metadata file). It includes the fields listed in Table 1. It is anticipated that updated versions will be made available periodically, as new sea surface iodide observations become available. The current iteration is termed Version 1.0, future iterations will be named sequentially (i.e. version 2.0 etc). The lead authors would be very pleased to be contacted regarding new or omitted iodide observations for inclusion in future iterations of the dataset.

## Technical Validation

Of the records included in our database, the majority (47/57) are described in peer-reviewed literature, and a further two are from PhD theses, and so their quality has already been subject to scientific scrutiny. Unpublished data sets made use of well-established analytical techniques, including the use of calibration standards and replicate analyses. In addition, the majority of data points were described in our earlier peer reviewed manuscript^12^, and were shown to have to a cohesive global distribution. The distribution of observations in the extended dataset continues to conform to this distribution (not shown), with concentrations remaining in the expected range (Fig. 2).

A very small number of unusually high concentration points (19 with iodide levels higher than 400 nM) are present in the data set. These are not representative of the overall iodide distribution, all being above the 98^th^ percentile and also defined as outliers under the Tukey definition^19^. Where present, these extreme outlier values have been subject to rigorous scrutiny and are believed to be real.

We have not evaluated the data set to look for systematic differences between measurement techniques, as method used and location (and hence iodide concentration) are not independent variables. In most cases, only a small number of geographically limited points are available for a given method (Table 2). As noted, more than half the observations have been made using the same CSSWV technique. The remainder have been analysed using a wide range of other approaches, including, for some of the earliest datasets, labour intensive ‘wet chemical’ procedures which have since been superseded. In particular, a large proportion of the Pacific measurements were made in between 1968 and 1970^20,21^ using a revised version of the Sugawara precipitation method^22^. The scarcity of more modern data from the Pacific limits comparisons, but we note that the range of this early Pacific data (3–168 nM) falls within that of the global data set, with a well-defined latitudinal distribution consistent with that observed overall. Regional concentrations (e.g. high latitudes, north Pacific^23^) are in agreement with those measured subsequently using different methods. Furthermore, the original data sources report vertical iodide profiles consistent in shape and magnitude with more recent measurements. Data obtained using the original, unmodified Sugawara method^24^ (1955) is not included, as this method is known to have poor performance^22^.

As described earlier, iodide observations are subject to non-negligible analytical uncertainty; we have reviewed the uncertainty estimation for each data set, and present this alongside the observations. As noted above, precision has usually been taken to represent method uncertainty. A variety of different methods have been used to estimate this, and so uncertainty magnitudes may not be directly comparable across all datasets.

## Usage Notes

For computational convenience, iodide concentrations and associated uncertainties are provided to one decimal place (units are nM for both). However, note that this does not usually reflect the precision of the data points correctly, as this is typically a few percent.

For the purposes of investigating large-scale trends and creating regional iodide parameterisations, it may be appropriate to exclude the very high outlier values noted in the preceding section. Similarly, a number of points are from relatively low salinity estuarine areas (e.g. the Skaggerak), and so may not be representative of true marine trends in iodine speciation.

Missing fields are shown as not a number (“NaN”) in the output data file.

## Data Availability

The Python code used to prepare the archived data, and to enable incorporation of any subsequent observational data files, has also been made permanently available (10.5281/zenodo.3271678)^25^.

## Online-only Table

**Online-only Table 1:** Summary of datasets included in this compilation.

#	Data_key	Geographical Location	n obs	Analytical method	Year(s)	Source	Reference
1	Chance_2007	Southern Ocean (Atlantic sector)	49	3	2004	Provided by originator	^51^
2	Chance_UnP_I	tropical east Atlantic	28	3	2007	Provided by originator	Unpublished
3	Elderfield_T_1980	Antarctic, Pacific, Atlantic	13	1	1976–1978	Publication	^30^
4	Truesdale_2003_I	Skagerrak	15	3	2001	Publication	^52^
5	Truesdale_2000	Atlantic	42	1	1996–1997	Publication	^31^
6	Truesdale_2001	Baltic	7	1	1999	Publication	^53^
7	Truesdale_2003_II	western Antarctic Peninsula	1	1	2001	Publication	^54^
8	Waite_2006	north Atlantic, Greenland & Norwegian Seas	13	3	2000	Publication	^55^
9	Wong_B_1977	Venezuela Basin, Cariaco Trench, Black Sea	3	6	~1975	Publication	^56^
10	Campos_1996	Atlantic & Pacific	14	3	1993–1994	Publication	^26^
11	Truesdale_B_2002	Benguela upwelling	1	1	1997	Publication	^57^
12	Tian_1995_1996	north west Mediterranean	20	3	1993–1994	Repository (PANGAEA)	^58–60^
13	Truesdale_1978	Indian, Atlantic, Irish Sea	6	1	1967, 1970, 1972, 1986	Publication	^61^
14	Jickells_1988	Sargasso Sea & Bermuda inshore	110	1	1984–1985	Provided by originator	^18^
15	Wong_B_1974	Argentine Basin, Angola Basin	3	1	1972	Publication	^27^
16	McTaggart_1994	east Australian coast	13	10	1991	Publication	^47^
17	Truesdale_U_2003	British east coast	3	1	1999	Publication	^62^
18	Wong_Z_2003	southern East China Sea	14	3	1992	Publication	^63^
19	Wong_Z_1992	southern Atlantic Bight	41	2	1987	Publication	^34^
20	Campos_1999	south Atlantic, Weddell Sea	17	3	1995	Repository (BODC)	^64^
21	Nakayama_1989_1985	north Pacific	13	5	1984, 1985	Publication	^23,41^
22	Farrenkopf_2002	Arabian Sea	3	3	1995	Repository (JGOFS)	^37,65^
23	Wong_1985	Orca Basin, Gulf of Mexico	1	2	1982	Publication	^66^
24	Ullman_1990	Mediterranean	7	3	1987	Publication	^38^
25	Woittiez_1991	Kau Bay, Indonesian coast	2	13	1985	Publication	^50^
26	Schwehr_S_2003	Gulf of Mexico, Galveston Bay	7	9	1999–2000	Publication	^46^
27	Tsunogai_1971	north Pacific	11	12	1970	Publication	^20^
28	Wong_C_1998	Chesapeake Bay	13	3	~1998?	Publication	^67^
29	Huang_2005	north Pacific	3	11	2003	Publication	^49^
30	Bluhm_2011	Southern Ocean (Atlantic sector)	23	3	2008	Publication	^68^
31	Wong_C_2008	Chesapeake Bay	9	3	1987, 1992, 1995	Publication	^69^
32	Hou_2007	North Sea	43	7	2005	Publication	^43^
33	Tsunogai_H_1971	Pacific	59	12	1968–1969	Publication	^21^
34	Liss_H_G_1973	southern Californian coast	6	2	1972	Publication	^35^
35	Luther_1988	north west Atlantic, Chesapeake bay	5	3	1987	Publication	^36^
36	Baker_UnP	Eastern, north & south Atlantic	16	3	2000	Provided by originator	Unpublished
37	Truesdale_J_2000	British coastal waters	185	1	1996	Repository (BODC)	^70^
38	Luther_UnP	south west Atlantic	1	3	1992–1993	Repository (BODC)	Unpublished
39	Ducklow_2018	western Antarctic Peninsula	19	3	2012	Provided by originator	^71^
40	Chance_UnP_II	tropical Atlantic	18	3	2011	Provided by originator	Unpublished
41	Hou_2013	Fukushima offshore	1	8	2011	Publication	^72^
42	Rue_1997	tropical North Pacific	1	4	1982	Publication	^40^
43	Chance_2010	western Antarctic Peninsula	61	3	2005–2008	Provided by originator	^16^
44	Luther_C_1991	Black Sea	2	3	1988	Publication	^73^
45	Wong_1977	Equatorial Atlantic	4	6	1974	Publication (thesis)	^74,75^
46	He_2013	north east Atlantic	34	7	2010	Provided by originator	^76^
47	He_2014	North Sea	12	7	2010	Provided by originator	^77^
48	Chance_2018_I	Indian Ocean	111	3	2017	Provided by originator	Unpublished
49	Chance_2018_II	Indian Ocean	7	3	2016	Provided by originator	Unpublished
50	Chance_2018_III	Indian Ocean	12	3	2016	Provided by originator	Unpublished
51	Chance_UnP_III	English Channel	23	3	2016–2018	Provided by originator	Unpublished
52	Croot_UnP	eastern Pacific	6	3	2008	Provided by originator	Unpublished
53	Cutter_2018	Peruvian upwelling	20	3	2013	Repository (BODC)	^11^
54	Zhou_2017	East China Sea	122	3	2014	Provided by originator	^78^
55	Campos_UnP	north west Atlantic	15	3	1992–1993	Provided by originator	Unpublished
56	Chance_UnP_IV	eastern Pacific	3	3	2018	Provided by originator	Unpublished
57	Chance_UnP_V	Benguela upwelling	6	3	2018	Provided by originator	Unpublished
